# XRRA1 Targets ATM/CHK1/2-Mediated DNA Repair in Colorectal Cancer

**DOI:** 10.1155/2017/5718968

**Published:** 2017-09-26

**Authors:** Wenjun Wang, Minzhang Guo, Xiaojun Xia, Chao Zhang, Yuan Zeng, Sipei Wu

**Affiliations:** ^1^State Key Laboratory of Respiratory Diseases, Guangzhou Institute of Respiratory Disease, The First Affiliated Hospital of Guangzhou Medical University, Guangzhou 510120, China; ^2^Experimental Research Department, Sun Yat-Sen University Cancer Center, Guangzhou 510060, China; ^3^Guangdong Lung Cancer Institute, Guangdong General Hospital and Guangdong Academy of Medical Sciences, Guangzhou 510080, China

## Abstract

X-ray radiation resistance associated 1 (XRRA1) has been found to regulate the response of human tumor and normal cells to X-radiation (XR). Although XRRA1 overexpression is known to be involved in cancer cell response to XR, there are no reports about whether the expression of XRRA1 in tumors can adjust radioresistance. It is widely known that cell cycle arrest could cause radioresistance. We found that blocked XRRA1 expression could lead to cell cycle G2/M arrest by the regulation of cyclin A, cyclin E, and p21 proteins in colorectal cancer (CRC) and expression of XRRA1 reduced cell cycle arrest and increased cell proliferation in CRC. However, whether regulation of the cell cycle by XRRA1 can influence radioresistance is poorly characterized. Correspondingly, DNA repair can effectively lead to radioresistance. In our study, when cancer cells were exposed to drugs and ionizing radiation, low expression of XRRA1 could increase the phosphorylation of DNA repair pathway factors CHK1, CHK2, and ATM and reduce the expression of *γ*-H2AX, which is believed to participate in DNA repair in the nucleus. Crucially, our results identify a novel link between XRRA1 and the ATM/CHK1/2 pathway and suggest that XRRA1 is involved in a DNA damage response that drives radio- and chemoresistance by regulating the ATM/CHK1/2 pathway.

## 1. Introduction

Colorectal cancer (CRC) is one of the most common human malignancies. In China, the incidence of CRC has increased gradually; incidence and death rates have risen to third and second place, respectively. Although surgery, chemotherapy, and radiotherapy are widely used in clinical treatment, the survival of patients is still very limited, with the five-year survival rate hovering between 25% and 30%. However, the important thing is to find the specific changes of tumor cells and to reduce the toxicity of normal cells while exerting stronger antitumor activity. This series of clinical problems has become a critical issue affecting the clinical gene therapy of tumors. Therefore, to further elucidate the pathogenesis of CRC and to find efficient, highly selective therapeutic targets are a major task in the field of gene therapy, and improving the efficacy of CRC treatment has important clinical significance.

DNA repair dysfunction is not only closely related to tumor development but also related to chemotherapy drug resistance. X-ray radiation resistance associated 1 (XRRA1) [1] is a new gene containing 11 exons that encodes a protein of 559 amino acids containing a leucine-rich repeat sequence, PEST sequence, and two tyrosine phosphorylation sites. The amino acid sequence of XRRA1 has great conservation between species. Located on chromosome 11q13.3, XRRA1 expression was significantly reduced in radiation-resistant HCT116Clone2_XRR CRC cell lines when compared with untreated HCT116Clone10 cells whereas in radiation-sensitive HCT116CloneK_XRS it was significantly upregulated. XRRA1 is mainly expressed in human and macaque testes; testicles are widely believed to be radiosensitive organs. In other tumors and normal tissues, XRRA1 is expressed and is upregulated in radiosensitive organs. The researchers observed that downregulated XRRA1 expression corresponded to the radiation resistance of HCT116Clone2_XRR cells. We believe that, in treatment for CRC, DNA damage repair is a common mechanism that causes cell resistance to therapy and that XRRA1 is a candidate model gene which is involved in this process. Therefore, we hypothesize that XRRA1 is likely to be related to tumor cell resistance to radiotherapy.

In the present study, we established XRRA1 knockdown and overexpression in CRC cell lines using stable transfection technology and validated the function of XRRA1 in the regulation of the cell cycle and proliferation. Moreover, we investigated the role of the XRRA1 gene in the mechanism of chemotherapy resistance. Further, our results identify a novel link between the XRRA1 and ATM/CHK1/2 pathway and suggest that XRRA1 is involved in the DNA damage response to drive radioresistance by regulating the ATM/CHK1/2 pathway. Our study provides a new candidate for CRC targeted drugs and individualized molecular targeted therapy.

## 2. Materials and Methods

### 2.1. Cell Culture and Transfection

HT29 and HCT116 cells were purchased from the American Type Culture Collection and cultured at 37°C with 5% CO_2_ in complete DMEM (Gibco, USA) supplemented with 10% fetal bovine serum and 2 mmol/L l-glutamine. Lipofectamine 2000 (Invitrogen Corporation) was used for transfection. Cell lines transfected with small hairpin RNA (shRNA) expression vectors were obtained after selective screening using G418 (800 *μ*g/mL).

### 2.2. Cell Proliferation Assays

The viability of HT29 and HCT116 cells was determined by assaying the reduction of 3-(4,5-dimethylthiazol-2-yl)-2,5-diphenyltetrazolium bromide (MTT; Beyotime Company, Shanghai, China) to formazan. Cells were seeded onto 96-well plates to analyze cell proliferation. A 5-ethynyl-2′-deoxyuridine (EdU) proliferation assay kit (Rui Bo, Guangzhou, China) was used to analyze the incorporation of EdU during DNA synthesis. Forty-eight hours after transfection, HT29 and HCT116 cells were exposed to 25 *μ*M EdU for 2 h at 37°C and then fixed in 4% PFA. After permeabilization with 0.5% Triton X, the cells were reacted with 1x Apollo reaction cocktail (RiboBio) for 30 min. Subsequently, the DNA of the cells was stained with Hoechst 33342 for 30 min and then photographed and counted under a fluorescence microscope (Nikon, japan).

### 2.3. Viral Vectors

Lentiviral plasmids (pLKO.1-puro) encoding sh-XRRA1 and sh-control were purchased from Sigma-Aldrich (Darmstadt, Germany). The cDNA of XRRA1 was cloned from human colorectal cell line HCT116 and ligated into the BamHI/AgeI restriction sites of GV358 (Genechem, Shanghai, China). Linearized vectors were recombined with target genes. The order of the vector elements was Ubi-MCS-3FLAG-SV40-EGFP-IRES-puromycin. The recombinant products were converted directly, and single clones were identified by PCR and sequence analysis. The recombinant lentiviral plasmid GV358-XRRA1 and the viral packaging auxiliary plasmids (Helper 1 and Helper 2) were transfected into 293T cells with three plasmids. The supernatant of transfected 48 h was collected according to the cell state after transfection.

When the HT29 and HCT116 cell grows to 80% confluence, add 5 *μ*l lentivirus for infection. The expression of fluorescence was observed by fluorescence microscope after 48 h. Then, add the medium containing G418 (150 *μ*g/mL) for screening. After 7 days, the culture medium containing G418 (75 *μ*g/mL) was used. After 2-3 weeks, positive clones were selected for extended culture.

### 2.4. Quantitative Real-Time PCR

Total RNA was extracted using TRIzol reagent (Invitrogen, Carlsbad, CA, USA) and the resulting mRNA was reverse transcribed into cDNA using 5x PrimeScript RT Master Mix (Takara Bio Group, Dalian, China). The reaction was conducted at 37°C for 15 min and 85°C for 5 s, according to the manufacturer's protocol. Quantitative PCR (qPCR) was performed using 2x SYBR Premix Ex Taq (Takara Bio Group) with a 7300 ABI Real-Time PCR System (Applied Biosystems, Foster City, CA, USA) under the following conditions: 95°C for 30 s, 95°C for 5 s, and 60°C for 30 s for 40 cycles. The relative mRNA levels were analyzed by the 2(^−ΔΔ^Ct) method with* GAPDH* as an internal control. Primers used for real-time PCR were as follows:

  XRRA1 forward 5′-TCAGGAATCTACAAGCTGGATGA-3′
  XRRA1 reverse 5′-CTGAACCACTAACCAGTGTCC-3′
  Cyclin E forward 5′-GGACACCATGGCCAAAATCGACAGG-3′
  Cyclin E reverse 5′-TTTCACTTGTCATGTCGTCCTTGTAGTCCG-3′
  Cyclin A2 forward 5′-AAGAGCGTGAAGATGCCCT-3′
  Cyclin A2 reverse 5′-GCATTTGGCTGTGAACTACAT-3′
  P21 forward 5′-TACTCCCCTGCCCTCAACAA-3′
  P21 reverse 5′-CGCTATCTGAGCAGCGCTCAT-3′
  GAPDH forward 5′-AATGGACAACTGGTCGTGGAC-3′
  GAPDH reverse 5′-CCCTCCAGGGGATCTGTTTG-3′

### 2.5. Immunofluorescence

Cells grown on glass slides were fixed with paraformaldehyde, permeabilized with Triton X-100, blocked with 1% BSA, and incubated with primary antibodies overnight. After washing with PBS, cells were incubated with fluorescence-labeled (Cy5) secondary antibody (Life Technologies, USA) for 45 min. Images were obtained using an inverted confocal laser scanning microscope (Olympus, Japan).

### 2.6. Antibodies and Immunoblotting

The following antibodies were used: anti-XRRA1 (sc-241747, Santa Cruz Biotechnology, USA), anti-phosphor CHK1 (number 2341; Cell Signaling Technology, USA), anti-total CHK1 (number 2345; Cell Signaling Technology, USA), anti-phosphor CHK2 (number 2666; Cell Signaling Technology, USA), anti-total CHK2 (number 2662; Cell Signaling Technology, USA), anti-phosphor ATM (number 5883; Cell Signaling Technology, USA), anti-total ATM (number 2873; Cell Signaling Technology, USA), anti-GAPDH, mouse IgG, and rabbit IgG (Santa Cruz Biotechnology, USA). Immunoblotting was performed as described previously [2].

### 2.7. Flow Cytometry Analysis

For cell cycle analysis, the cells were separated into single cells by digestion and then collected by centrifugation. The supernatant was discarded and the cells were washed twice with precooled PBS, 3 mL of precooled 70% ethanol was added to the cell pellet, and cells were fixed overnight at 4°C. The cells were collected by centrifugation (5 min/1000 rpm) and washed twice with 3 mL PBS. Then, 500 *μ*L PBS containing 50 *μ*g/mL PI, 100 *μ*g/mL RNase A, and 0.2% Triton X-100 was added and cells were incubated in the dark at 4°C for 30 min before being analyzed by FACSCalibur (BD Biosciences, USA).

Cell apoptosis was analyzed using an Apoptosis Detection Kit (Life Technologies, USA) with reference to the instructions.

### 2.8. Statistical Analysis

Data are expressed as the mean value ± standard deviation of at least triplicate independent determinations of the quantitative assays in this study. Significant differences were analyzed using Student's *t*-test. A value *P* < 0.05 was considered statistically significant. Spearman's rank correlation coefficient was calculated using SPSS software.

## 3. Results

### 3.1. Detection of XRRA1 Lentivirus Transfection Efficiency

**Figure 1:**
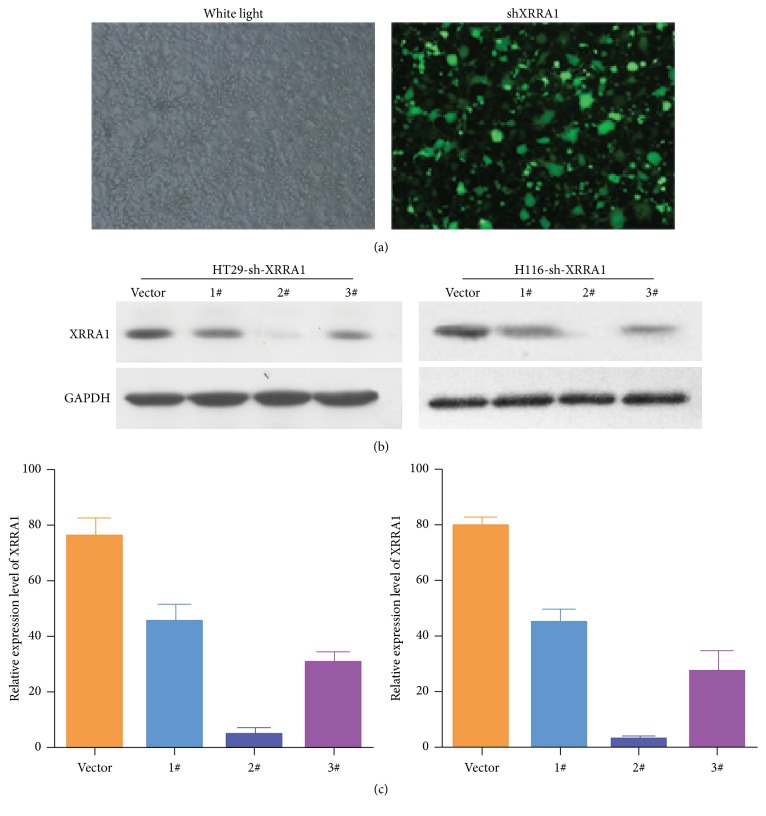
*Transfection efficiency of the sh-XRRA1 lentivirus*. (a) The transfection efficiency of sh-XRRA1 was observed under fluorescent microscopy. (b) Western blot analysis of the expression of XRRA1 in HT29 and HCT116 cell lines after shRNA knockdown by the sh-XRRA1 vector. (c) Quantitative real-time PCR analysis showed the expression of XRRA1 in HT29 and HCT116 cell line after XRRA1 was downregulated.

After a 48 h incubation, the green fluorescent proteins carried by the sh-XRRA1-lentiviral plasmid were observed under a fluorescence microscope (Figure 1(a)). To understand further the role of XRRA1 in regulating cell proliferation in cancer, we depleted XRRA1 expression by using XRRA1 shRNA, three XRRA1 shRNAs were constructed. After HT29 and HCT116 CRC cell lines were infected, western blot and quantitative real-time PCR were used to examine the inhibitory effect of shRNA on XRRA1. We found that sh-XRRA1 2# was more effective at blocking expression than the others (Figures 1(b) and 1(c)).

### 3.2. The Expression of XRRA1 Influences the Proliferation of HT29 and HCT116 Cell Lines

**Figure 2:**
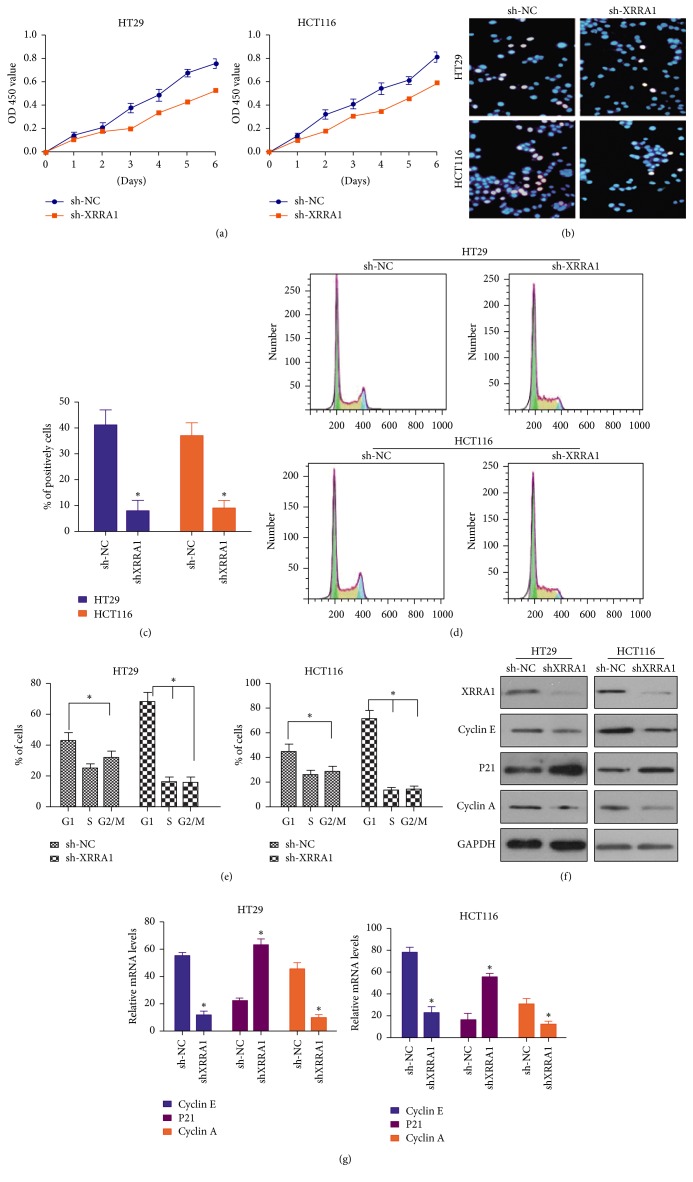
*Downregulation of XRRA1 expression inhibits cell proliferation in HT29 and HCT116 cell lines*. (a) MTT assay of HT29 and HCT116 cell proliferation after downregulation of XRRA1 expression by the sh-XRRA1 vector. (b) The percentages of cells incorporated by EdU were determined by examining at least 200 cells per sample in multiple fields. Data in C are averages of three repeats. (d) Cell cycle analysis by flow cytometry of XRRA1 at low expression in HT29 and HCT116 cells. Data in E are the averages of three repeats. (f) Western blot was used to determine the expression of cyclin E, p21, and cyclin A at the protein level after sh-XRRA1 was infected into HT29 and HCT116 cells. (g) Quantitative real-time PCR was used to determine the expression of cyclin E, p21, and cyclin A at the mRNA level after sh-XRRA1 was infected into HT29 and HCT116 cells.

**Figure 3:**
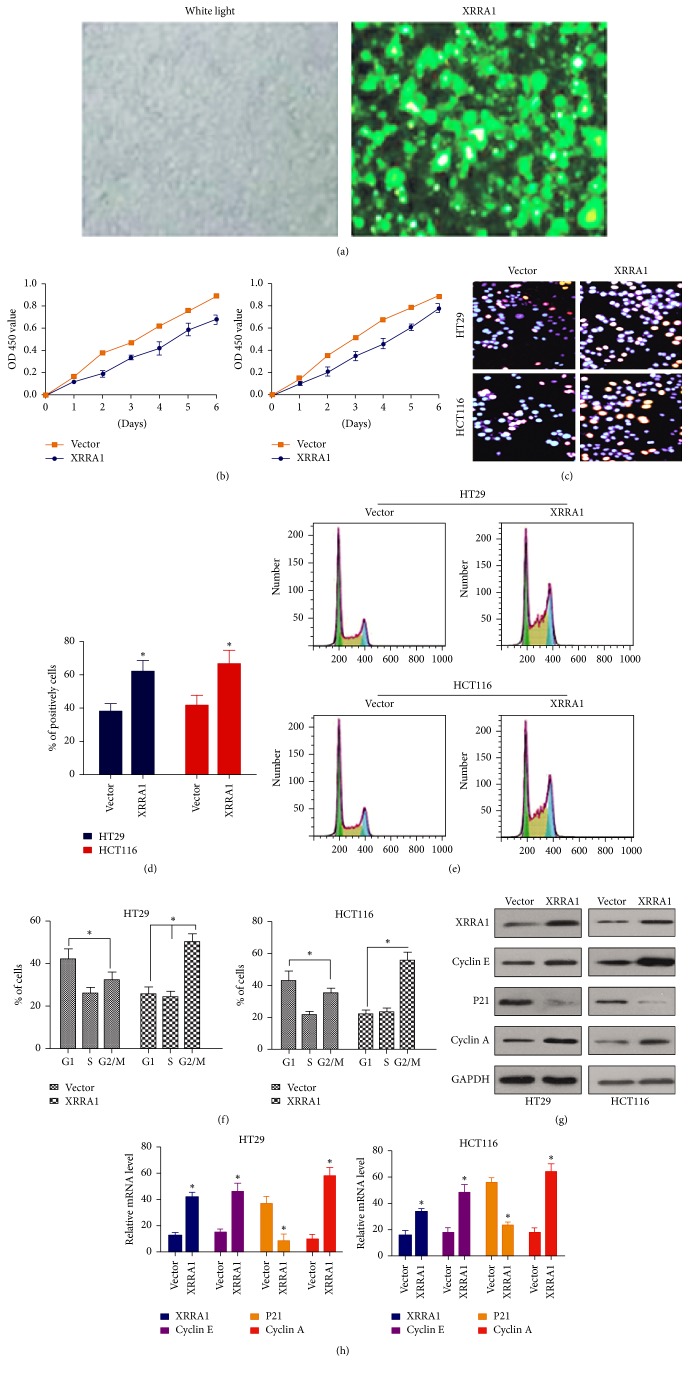
*Overexpression of XRRA1 induces cell proliferation in HT29 and HCT116 cell lines*. (a) The infection efficiency of XRRA1 was observed by fluorescence microscopy. (b) MTT assay shows that overexpression of XRRA1 in HT29 and HCT116 cells increased cell growth. (c) The percentage of cells that incorporated EdU was determined by examining at least 200 cells per sample in multiple fields. Data in (d) are averages of three repeats. (e) Cell cycle analysis of XRRA1 overexpression in HT29 and HCT116 cells by flow cytometry. Data in (f) are averages of three repeats. (g) Western blot analyses of cyclin E, p21, and cyclin A in HT29 and HCT116 cells after XRRA1 lentivirus infection. (h) Quantitative real-time PCR analysis of cyclin E, p21, and cyclin A in HT29 and HCT116 cells after XRRA1 lentivirus infection.

To determine whether XRRA1 expression could influence cell proliferation in CRC, an MTT assay was used to compare cell proliferation in CRC cell lines HT29 and HCT116. We found that low XRRA1 expression significantly decreased cell proliferation in CRC cells compared with empty vector-transfected cells (Figure 2(a)). Contrarily, overexpression of XRRA1 promotes HT29 and HCT116 cell proliferation (Figures 3(a) and 3(b)). A BrdU labeling assay was performed in HCT116 and HT29 after XRRA1 was blocked by sh-XRRA1. We confirmed that sh-XRRA1 could decrease CRC cell proliferation (Figures 2(b) and 2(c)). However, overexpression of XRRA1 by infected GFP-XRRA1 lentivirus was shown to increase CRC cell proliferation (Figures 3(c) and 3(d)).

### 3.3. XRRA1 Controls the Cell Cycle by Regulating Cyclin A, Cyclin E, and p21 Proteins

Our results found that XRRA1 can increase cancer cell proliferation because the cell cycle was related to cell proliferation, to confirm whether the influence of cancer cells proliferation by XRRA1 was due to cell cycle regulation. We inhibited XRRA1 expression by XRRA1 shRNA and also overexpressed XRRA1 by GFP-XRRA1 lentivirus infection; flow cytometric analysis was used to check changes of the cell cycle. We found that XRRA1 overexpression could arrest both the CRC cell lines HT29 and HCT116 in G2/M; simultaneously, blocking the expression of XRRA1 by shRNA resulted in G1 arrest (Figures 2(d), 2(e), 3(e), and 3(f)). It is widely known that cycle arrest results in the inhibition of cell proliferation. Cyclin D1 and cyclin A are regulated by p21 to influence the cell cycle and cell proliferation [1, 3]. Overexpression of cyclin A and cyclin E could induce G_1_ arrest [4], whereas p21 mediated G2-phase cell cycle arrest [5]. To determine the molecular mechanism that contributed to enhanced cell proliferation by XRRA1, western blots were used to elucidate the activation of the key cell cycle signaling pathways related to XRRA1 expression. As shown in Figures 3(g) and 3(h), overexpression of XRRA1 could activate cyclin A and cyclin E but decrease expression of p21. Consistently, downregulation of XRRA1 by shRNA decreased the expression of cyclin A, cyclin E, and p21 proteins (Figures 2(f) and 2(g)). Taken together our results suggest that XRRA1 regulates the cell cycle by targeting cyclin A, cyclin E, and p21.

In our study, we found that overexpression of XRRA1 resulted in dense labeling of proliferating cells, but depletion of XRRA1 expression reduces cell proliferation. These results suggest that XRRA1 can drive cancer proliferation in CRC.

### 3.4. Ionizing Radiation Downregulates the Expression of XRRA1 in a Dose- and Time-Dependent Manner in CRC Cells

**Figure 4:**
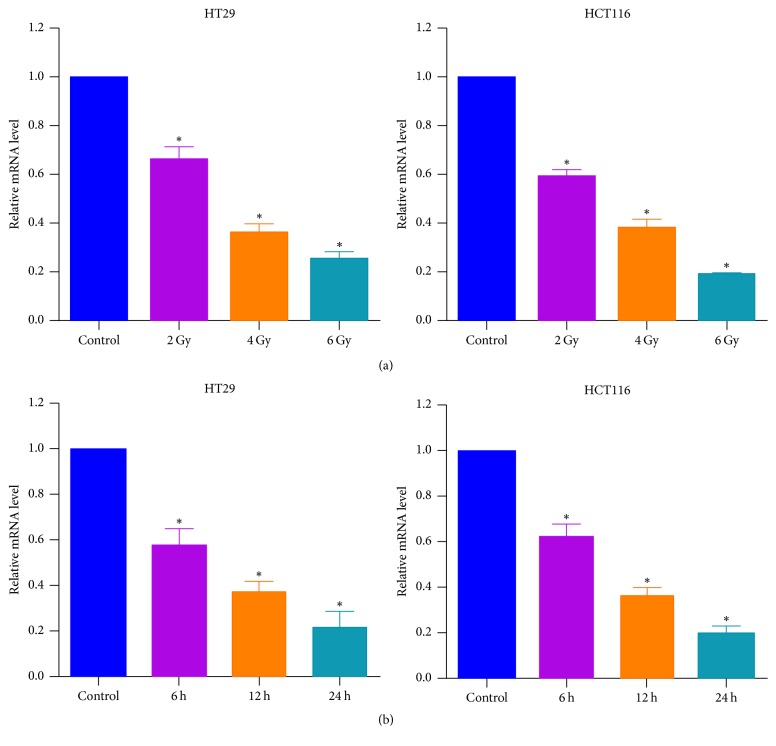
*Ionizing radiation downregulated the expression of XRRA1 at mRNA and protein levels in CRC cells*. (a) Cells were treated with different irradiation doses for 24 h and assessed for relative mRNA expression of XRRA1 using quantitative real-time PCR. Error bars indicate standard deviations of three independent experiments. (b) The cells were treated with 6 Gy at different times and assessed for the relative mRNA expression of XRRA1 using quantitative real-time PCR. The average expression of the target gene was normalized to the corresponding value for GAPDH and expressed as the fold change compared with controls.

The expression of XRRA1 mRNA was determined in CRC cells after treatment with ionizing radiation (IR) at 2, 4, and 6 Gy for 24 h using a real-time PCR assay. As shown in Figure 4(a), XRRA1 mRNA levels in IR-treated cells were significantly decreased in a dose-dependent manner. To further explore the time effect relationship between IR treatment and XRRA1 expression, we measured XRRA1 expression at mRNA levels after treatment with IR at various time points (6, 12, and 24 h). The results show that IR decreased the expression of XRRA1 at the mRNA levels in CRC cells in a time-dependent manner (Figure 4(b)).

### 3.5. Downregulated Expression of XRRA1 in CRC Cells Can Regulate the ATM/CHK1/2 Pathway to Mediated DNA Damage Response after IR Treatment

**Figure 5:**
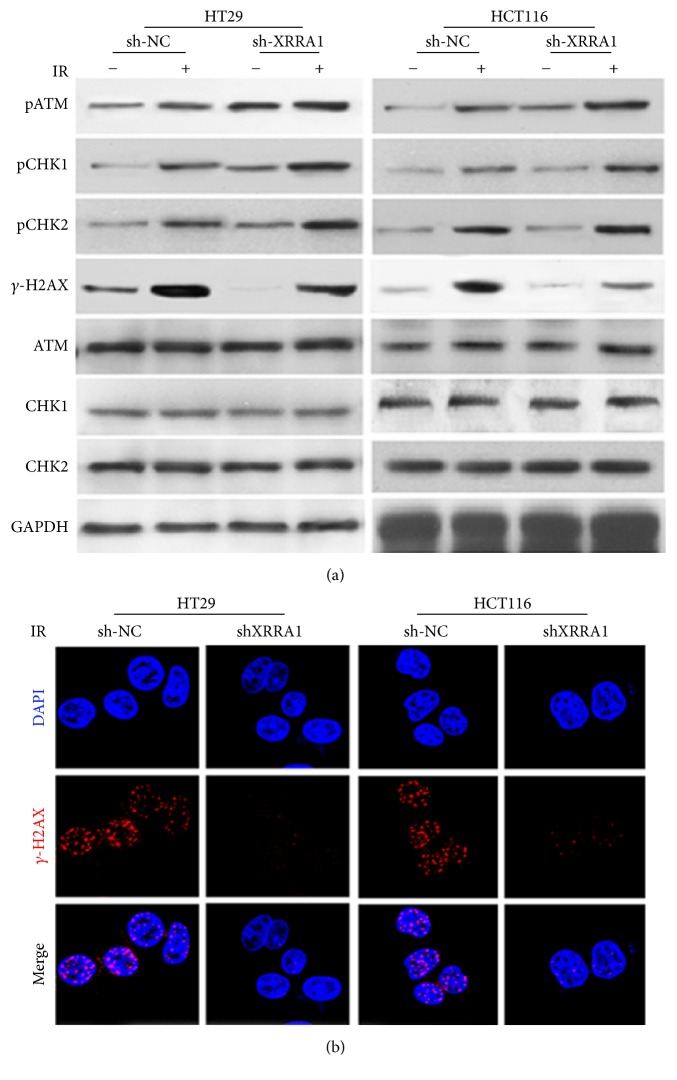
*Downregulated expression of XRRA1 in CRC cells regulates ATM/CHK1/2 pathway to mediated DNA damage response after ionizing radiation*. (a) Western blot analyses of pCHK1, pCHK2, and r-H2AX in HT29 and HCT116 cells were irradiated for 6 hours. (b) Immunofluorescence staining of *γ*-H2AX to evaluate the expression of sh-XRRA1 in HT29 and HCT116 cells was irradiated for 6 hours.

DNA repair pathways also play an important role in DNA-damaging cytotoxic therapy resistance, as well as radiation. Thus, we determined the functional role of CHK1/CHK2 DNA repair pathways in XRRA1. Our studies showed that, after 6 h of radiation, low expression of XRRA1 increased the phosphorylation of checkpoint kinase-1/2 (CHK1/2), which has been reported as an important kinase with vital roles in cell cycle arrest and DNA damage response, and the activation of ATM, a protein that was also inhibited by the low expression of XRRA1 [6]. Interestingly, following IR, the expression of *γ*-H2AX, a variant of the histone H2A family that is believed to participate in DNA repair in the nucleus, was decreased with the low expression of XRRA1 more than in control cells (Figure 5(a)). Then we used immunofluorescence detection of *γ*-H2AX function and found that XRRA1 expression was inhibited following irradiation and the expression of H2AX was markedly decreased. This suggests that the degree of DNA damage is weakened (Figure 5(b)); carboplatin (CBP) and capecitabine (CAPE) are common first-line chemotherapeutic agents in CRC treatment. In order to determine whether the expression of XRRA1 can affect DNA damage induced by chemotherapy, we first inhibited the expression of XRRA1 in HT29 and H116 cell lines. CBP and CAPE were then used to detect the expression of *γ*-H2AX after 24 h. HT29 and H116 cell lines were treated with CBP (IC50, 37.1 ± 1.4 *μ*g/mL) and CAPE (IC50, 16.1 ± 4.5 *μ*g/mL). After 24 h, immunofluorescence assay revealed that the expression of *γ*-H2AX was lower in CRC cells (Supplementary Figure 1 in Supplementary Material available online at https://doi.org/10.1155/2017/5718968). Overall, XRRA1 could prevent DNA damage repair and increase DNA damage by inhibition of ATM/CHK1/2 in CRC cells.

## 4. Discussion

To the best of our knowledge, this is the first study that is mainly focused on XRRA1 biological function in cancer. Our results suggest that up- or downexpression of XRRA1 can affect cell proliferation. Expression of XRRA1 is also closely related to the sensitivity of tumor radiotherapy.

The XRRA1 gene comprises 11 exons and spans 64 kb on chromosome 11q13.3. Human XRRA1 cDNA is 1987 nt long and encodes a protein of 559 aa. Mesak et al. [1] detected XRRA1 expression in normal tissues/organs and cells, as well as in various cancer cell types (such as breast cancer, glioma, melanoma, lung cancer, and neuroblastoma) by RT-PCR and found that XRRA1 was present in all cell types, although the expression level was variable. To understand further the possible function of the XRRA1 gene, we overexpressed XRRA1 in six different tumor cell lines and found significantly increased cell proliferation compared with the control. Cell proliferation is an important indicator in the evaluation of cell activity and metabolic, physiological, and pathological conditions. The EdU cell proliferation assay does not require severe DNA denaturation and can better protect the cell morphology, DNA overall structure, and the antigen recognition site of the cell. Using EdU detection, we found that reduced expression of XRRA1 in HT29H and HCT116 can affect cell proliferation. As shown in Figures 3(b) and 3(c), overexpression of XRRA1 also can affect cell proliferation. Cell cycle was detected by flow cytometry; XRRA1 on cell proliferation is mainly the cell cycle arrest in G1 phase. Overexpression of XRRA1 induces cyclin E and cyclin A and reduces p21 expression. P21, located downstream of the p53 gene, is an important member of the family of cyclin kinase inhibitors [4]. One mechanism by which tumor cells develop resistance to cytotoxic agents and radiation is related to apoptosis resistance. P21 and p53 may constitute G1 cell cycle checkpoints, which can lead to chemotherapy and radiotherapy resistance, thus affecting the therapeutic effect. Previous studies reported that some drugs, mircoRNA, and proteins display an anticancer effect on regulation of the G0/G1 phase of the cell cycle and G0/G1 arrest implies chemoresistance in certain types of tumor [7–9]. As first-line chemotherapy, carboplatin (CBP) and capecitabine (CAPE) mediate a G1 phase prolongation to conduct chemoresistance [10, 11]. Our results found that inhibition of XRRA1 could decrease the expression of *γ*-H2AX after CBP and CAPE treatment, indicating that XRRA1 could work as a factor to forecast the response of chemotherapy in CRC.

Moreover, we proved that XRRA1 impact on drug sensitivity was mainly activated through the DNA damage checkpoint reaction. Previous reports [12–15] suggest that cell cycle checkpoint kinases CHK1 and CHK2 play an important role after DNA damage by drugs, IR, and ultraviolet (UV) caused by the phosphorylation and activation of ATM and ATR and in S and G_2_ phase checkpoint regulation. CHK1 and CHK2 are important ATM and ATR substrates; the CHK1 checkpoint is necessary for S phase. CHK1 and CHK2 are important substrates for ATM and ATR. After DNA damage, ATR/ATM made CHK1 and CHK2 acquire kinase activity and then phosphorylated the downstream CDC25 family and other substrates. After UV and IR-induced DNA damage or replication arrest, ATR/ATM phosphorylation activates CHK1 [16]. CDC25A phosphorylation of the serine 123, serine 178, serine 278, and serine 292 accelerated CDC25A degradation; inhibition of cyclin E/CDK2 kinase complex causes S phase arrest [17]. CHK2 phosphorylation activates the regulation of many proteins, such as NBSl, E2F1, 53BPl, MDCl, MRK, and RPAl, to promote an ATM on CHK2-T68 phosphorylation [18].

In summary, our study demonstrates that XRRA1 is associated with sensitivity to radiotherapy in CRC. Our data supports the finding that XRRA1 can promote the proliferation of CRC cells. In addition, XRRA1 can regulate cell cycle-related proteins. We also demonstrate that overexpression of XRRA1 increases apoptosis rate and reduced colony formation efficiency. Lastly, our results highlight that after DNA damage, the downregulation of XRRA1 expression enhanced ATM activity to activate CHK1 and CHK2, thereby regulating radiotherapy sensitivity of CRC cells.

## Supplementary Material

sFigure 1: Down-regulated expression of XRRA1 in CRC cells can reduce the expression of γ-H2AX after chemotherapeutic agents.
