# Corrigendum to “XRRA1 Targets ATM/CHK1/2-Mediated DNA Repair in Colorectal Cancer”

**DOI:** 10.1155/2021/3030267

**Published:** 2021-02-19

**Authors:** Wenjun Wang, Minzhang Guo, Xiaojun Xia, Chao Zhang, Yuan Zeng, Sipei Wu

**Affiliations:** ^1^State Key Laboratory of Respiratory Diseases, Guangzhou Institute of Respiratory Disease, The First Affiliated Hospital of Guangzhou Medical University, Guangzhou 510120, China; ^2^Experimental Research Department, Sun Yat-Sen University Cancer Center, Guangzhou 510060, China; ^3^Guangdong Lung Cancer Institute, Guangdong General Hospital and Guangdong Academy of Medical Sciences, Guangzhou 510080, China

In the article titled “XRRA1 Targets ATM/CHK1/2-Mediated DNA Repair in Colorectal Cancer” [1], there were errors in the reported sequences. It was raised to our attention [2] that the article includes mistargeted primer sequences as follows:

(a) The reverse primer for cyclin E, TTTCACTTGTCATGTCGTCCTTGTAGTCCG, does not target cyclin E despite being reported to do so in previous studies [3, 4]

(b) The primers given for P21, TACTCCCCTGCCCTCAACAAG and CGCTATCTGAGCAGCGCTCAT, actually target TP53

(c) The primers given for GADPH, AATGGACAACTGGTCGTGGAC and CCCTCCAGGGGATCTGTTTG, actually target glyceraldehyde-3-phosphate dehydrogenase, spermatogenic. This is a paralog of GADPH that is only expressed in high levels in the testes [5]

The authors say they used the correct primers and provided purchase orders (see the supplementary materials (available here)), but incorrectly included primers used in their other research when preparing the article. The correct primer sequences are shown below:

- XRRA1 forward 5′-TCAGGAATCTACAAGCTGGATGA-3′
- XRRA1 reverse 5′-CTGAACCACTAACCAGTGTCC-3′
- Cyclin E forward 5′-GCCAGCCTTGGGACAATAATG-3′
- Cyclin E reverse 5′-CTTGCACGTTGAGTTTGGGT-3′
- Cyclin A2 forward 5′-CGCTGGCGGTACTGAAGTC-3′
- Cyclin A2 reverse 5′-GAGGAACGGTGACATGCTCAT-3′
- P21 forward 5′-CGATGGAACTTCGACTTTGTCA-3′
- P21 reverse 5′-GCACAAGGGTACAAGACAGTG-3′
- GAPDH forward 5′-ACAACTTTGGTATCGTGGAAGG-3′
- GAPDH reverse 5′-GCCATCACGCCACAGTTTC-3′

## Supplementary Materials

Supplementary Materials Purchase orders for the primers from Tianyi Huiyuan.
