# Supplementary material for: XRRA1 Targets ATM/CHK1/2-Mediated DNA Repair in Colorectal Cancer
Source: Biomed Res Int. 2017 Sep 26;2017:5718968. doi: 10.1155/2017/5718968 (PMC5634579; doi:10.1155/2017/5718968)

sFigure 1. Down-regulatedexpression of XRRA1 in CRC cells can reduce the expression ofγ-H2AXafter chemotherapeutic agents.

A. Immunofluorescence staining of γ-H2AX to evaluate the expression of sh-XRRA1 in HT29 and HCT116 cells were treated with capecitabine(CAPE) and carboplatin(CBP) for 24 h

SFigure 1

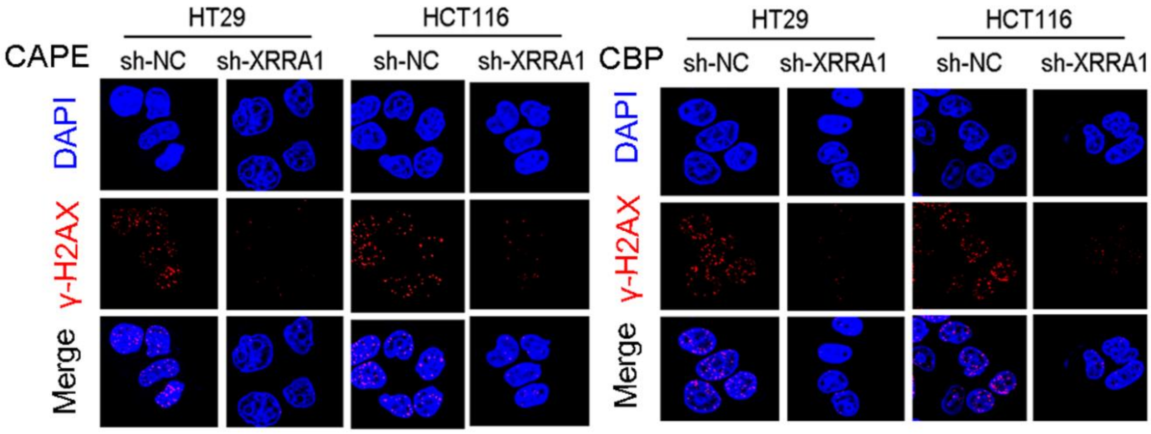

Supplement: Supplementary file 1 — sFigure 1: Down-regulated expression of XRRA1 in CRC cells can reduce the expression of γ-H2AX after chemotherapeutic agents. [file 5718968.f1.pdf]
