# Supplementary material for: Corrigendum to “XRRA1 Targets ATM/CHK1/2-Mediated DNA Repair in Colorectal Cancer”
Source: Biomed Res Int. 2021 Feb 19;2021:3030267. doi: 10.1155/2021/3030267 (PMC7935600; doi:10.1155/2021/3030267)
Supplement: Supplementary Materials — Purchase orders for the primers from Tianyi Huiyuan. [file 3030267.f1.pdf]

天一辉远 TIANYI HUIYUAN

广州天一辉远基因科技有限公司 DNA合成报告单

客户姓名： 王文君 广州医科大学 订单号： SY0110915 订购日期： 2015-12-5

流水号	引物名称	引物序列(5-3)	碱基数	总OD	OD/管	分装管数	纯化方式	MW	Tm/0.05M (°C)	GC%	nmol/OD	nmol/tube	ug/tube	Add H_2O Volumn for 100uM (ul)
011180280	XRRA1 forward①	TCAGGAATCTACAAGCTGGATGA	23	2	2	1	PAGE	7096.64	56.21	43.00	3.75	7.50	53.24	75.02
011180281	XRRA1 reverse①	CTGAACCACTAACCAGTGTCC	21	2	2	1	PAGE	6335.24	57.59	52.00	4.52	9.05	57.31	90.46
011180282	XRRA1 forward②	CTCGGCGGGAAAATCAGGT	19	2	2	1	PAGE	5877.84	57.09	58.00	4.66	9.32	54.81	93.24
011180283	XRRA1 reverse②	TGGAAACGTGTGAAATGCCTC	21	2	2	1	PAGE	6470.24	55.63	48.00	4.24	8.48	54.88	84.82
011180284	XRRA1 forward③	CTGGATCTCGCATTTAATGGCA	22	2	2	1	PAGE	6725.44	55.94	45.00	4.28	8.56	57.58	85.62
011180285	XRRA1 reverse③	TTGCTTGTCAGCGATGTTACA	21	2	2	1	PAGE	6427.24	53.68	43.00	4.55	9.10	58.51	91.03
011180286	XRRA1 forward④	AACCTCAAGAAGAAGCCC	18	2	2	1	PAGE	5470.64	52.24	50.00	4.74	9.48	51.88	94.83
011180287	XRRA1 reverse④	AACTCAAAAGAAGTCGCC	18	2	2	1	PAGE	5485.64	49.97	44.00	4.71	9.42	51.65	94.16
011180288	XRRA1 forward ⑤	ACTGCCTCCCATCTGCTC	18	2	2	1	PAGE	5346.64	56.80	61.00	6.10	12.20	65.24	122.03
011180289	XRRA1 reverse⑤	CCGTTGGTGGTTTCTTTT	18	2	2	1	PAGE	5493.64	49.97	44.00	5.94	11.88	65.25	118.76
011180290	Cyclin E forward ①	AAGGAGCGGGACACCATGA	19	2	2	1	PAGE	5895.84	57.09	58.00	4.39	8.78	51.79	87.83

感谢您选择天一为您提供合成服务，请您在使用前参考背面的相关说明！

扫描全能王 创建

流水号	引物名称	引物序列(5-3)	碱基数	总OD	OD/管	分装管数	纯化方式	MW	Tm/0.05M (°C)	GC%	nmol/OD	nmol/tube	ug/tube	Add H_2O Volume for 100uM (ul)
011180291	Cyclin E reverse ①	ACGGTCACGTTTGCCTTCC	19	2	2	1	PAGE	5730.84	57.09	58.00	5.51	11.02	63.15	110.19
011180292	Cyclin E forward ②	GCCAGCCTTGGGACAATAATG	21	2	2	1	PAGE	6455.24	57.59	52.00	4.27	8.54	55.10	85.36
011180293	Cyclin E reverse ②	CTTGCACGTTGAGTTTGGGT	20	2	2	1	PAGE	6170.04	55.30	50.00	4.88	9.76	60.20	97.56
011180294	Cyclin A2 forward ①	CGCTGGCGGTACTGAAGTC	19	2	2	1	PAGE	5844.84	59.25	63.00	5.01	10.01	58.51	100.10
011180295	Cyclin A2 reverse ①	GAGGAACGGTGACATGCTCAT	21	2	2	1	PAGE	6495.24	57.59	52.00	4.19	8.38	54.42	83.79
011180296	Cyclin A2 forward ②	TGGAAAGCAAACAGTAAACAGCC	23	2	2	1	PAGE	7083.64	56.21	43.00	3.55	7.09	50.24	70.92
011180297	Cyclin A2 reverse ②	GGGCATCTTCACGCTCTATTT	21	2	2	1	PAGE	6363.24	55.63	48.00	4.83	9.65	61.42	96.53
011180298	P21 forward①	TGTCCGTCAGAACCCATGC	19	2	2	1	PAGE	5748.84	57.09	58.00	5.14	10.27	59.05	102.72
011180299	P21 reverse①	AAAGTCGAAGTTCCATCGCTC	21	2	2	1	PAGE	6390.24	55.63	48.00	4.41	8.81	56.30	88.11
011180300	P21 forward②	CGATGGAACTTCGACTTTGTCA	22	2	2	1	PAGE	6725.44	55.94	45.00	4.28	8.56	57.58	85.62
011180301	P21 reverse②	GCACAAGGGTACAAGACAGTG	21	2	2	1	PAGE	6513.24	57.59	52.00	3.97	7.94	51.71	79.40

扫描全能王 创建

流水号	引物名称	引 物 序 列(5-3)	碱基数	总OD	OD/管	分装管数	纯化方式	MW	Tm/0.05M (^{0}C)	GC%	nmol/OD	nmol/tube	ug/tube	Add H_2O Volumn for 100uM (ul)
011180302	P21 forward③	TGGGGATGTCCGTCAGAA	18	2	2	1	PAGE	5579.64	54.52	56.00	4.99	9.97	55.63	99.70
011180303	P21 reverse③	CCCGTGGGAAGGTAGAGC	18	2	2	1	PAGE	5589.64	59.08	67.00	4.95	9.90	55.34	99.01
011180304	GAPDH forward①	GGAGCGAGATCCCTCCAAAAT	21	2	2	1	PAGE	6424.24	57.59	52.00	4.23	8.46	54.33	84.57
011180305	GAPDH reverse①	GGCTGTTGTCATACTTCTCATGG	23	2	2	1	PAGE	7036.64	58.00	48.00	4.31	8.62	60.63	86.17
011180306	GAPDH forward②	ACAACTTTGGTATCGTGGAAGG	22	2	2	1	PAGE	6814.44	55.94	45.00	4.02	8.03	54.73	80.32
011180307	GAPDH reverse②	GCCATCACGCCACAGTTTC	19	2	2	1	PAGE	5708.84	57.09	58.00	5.25	10.51	60.00	105.10

扫描全能王 创建
